# Fully-automated left ventricular mass and volume MRI analysis in the UK Biobank population cohort: evaluation of initial results

**DOI:** 10.1007/s10554-017-1225-9

**Published:** 2017-08-23

**Authors:** Avan Suinesiaputra, Mihir M. Sanghvi, Nay Aung, Jose Miguel Paiva, Filip Zemrak, Kenneth Fung, Elena Lukaschuk, Aaron M. Lee, Valentina Carapella, Young Jin Kim, Jane Francis, Stefan K. Piechnik, Stefan Neubauer, Andreas Greiser, Marie-Pierre Jolly, Carmel Hayes, Alistair A. Young, Steffen E. Petersen

**Affiliations:** 1 Department of Anatomy and Medical Imaging, Faculty of Medical and Health Sciences, University of Auckland, 85 Park Road, Auckland, 1142 New Zealand; 2 William Harvey Research Institute, NIHR Cardiovascular Biomedical Research Centre at Barts, Queen Mary University of London, Charterhouse Square, London, EC1M 6BQ UK; 3 Division of Cardiovascular Medicine, Radcliffe Department of Medicine, University of Oxford, Oxford, UK; 4 Siemens Healthcare GmbH, Erlangen, Germany; 5 Siemens Medical Solutions USA, Inc., Princeton, NJ USA

**Keywords:** Ventricular function, Automated analysis, UK Biobank

## Abstract

UK Biobank, a large cohort study, plans to acquire 100,000 cardiac MRI studies by 2020. Although fully-automated left ventricular (LV) analysis was performed in the original acquisition, this was not designed for unsupervised incorporation into epidemiological studies. We sought to evaluate automated LV mass and volume (Siemens *syngo* InlineVF versions D13A and E11C), against manual analysis in a substantial sub-cohort of UK Biobank participants. Eight readers from two centers, trained to give consistent results, manually analyzed 4874 UK Biobank cases for LV end-diastolic volume (EDV), end-systolic volume (ESV), stroke volume (SV), ejection fraction (EF) and LV mass (LVM). Agreement between manual and InlineVF automated analyses were evaluated using Bland–Altman analysis and the intra-class correlation coefficient (ICC). Tenfold cross-validation was used to establish a linear regression calibration between manual and InlineVF results. InlineVF D13A returned results in 4423 cases, whereas InlineVF E11C returned results in 4775 cases and also reported LVM. Rapid visual assessment of the E11C results found 178 cases (3.7%) with grossly misplaced contours or landmarks. In the remaining 4597 cases, LV function showed good agreement: ESV −6.4 ± 9.0 ml, 0.853 (mean ± SD of the differences, ICC) EDV −3.0 ± 11.6 ml, 0.937; SV 3.4 ± 9.8 ml, 0.855; and EF 3.5 ± 5.1%, 0.586. Although LV mass was consistently overestimated (29.9 ± 17.0 g, 0.534) due to larger epicardial contours on all slices, linear regression could be used to correct the bias and improve accuracy. Automated InlineVF results can be used for case-control studies in UK Biobank, provided visual quality control and linear bias correction are performed. Improvements between InlineVF D13A and InlineVF E11C show the field is rapidly advancing, with further improvements expected in the near future.

## Introduction

UK Biobank is a large prospective cohort study designed to assess the determinants of diseases of middle and old age [1]. Initial data collection in 500,000 participants, including genetic, physical and functional measures, was completed in 2010. Participants will be followed for 20 years, enabling nested case-control studies to assess exposures and pre-existing characteristics in the development of disease and the effect of treatment. In 2013, an imaging extension was initiated with the goal of imaging 100,000 UK Biobank participants by 2020 [2]. The imaging studies include a 20 min cardiovascular magnetic resonance (CMR) examination, to assess cardiac phenotypes including ventricular function [3]. However, analysis of ventricular function parameters in 100,000 cases is impractical using current manual methods, which require drawing the ventricular boundaries at end-diastole (ED) and end-systole (ES) [4]. Also, manual assessment requires substantial training and is subject to inter-observer and inter-center variation [5]. Large-scale CMR studies, such as UK Biobank, therefore present substantial challenges and opportunities for epidemiological analysis of cardiac phenotypes [6–9].

**Fig. 1:**
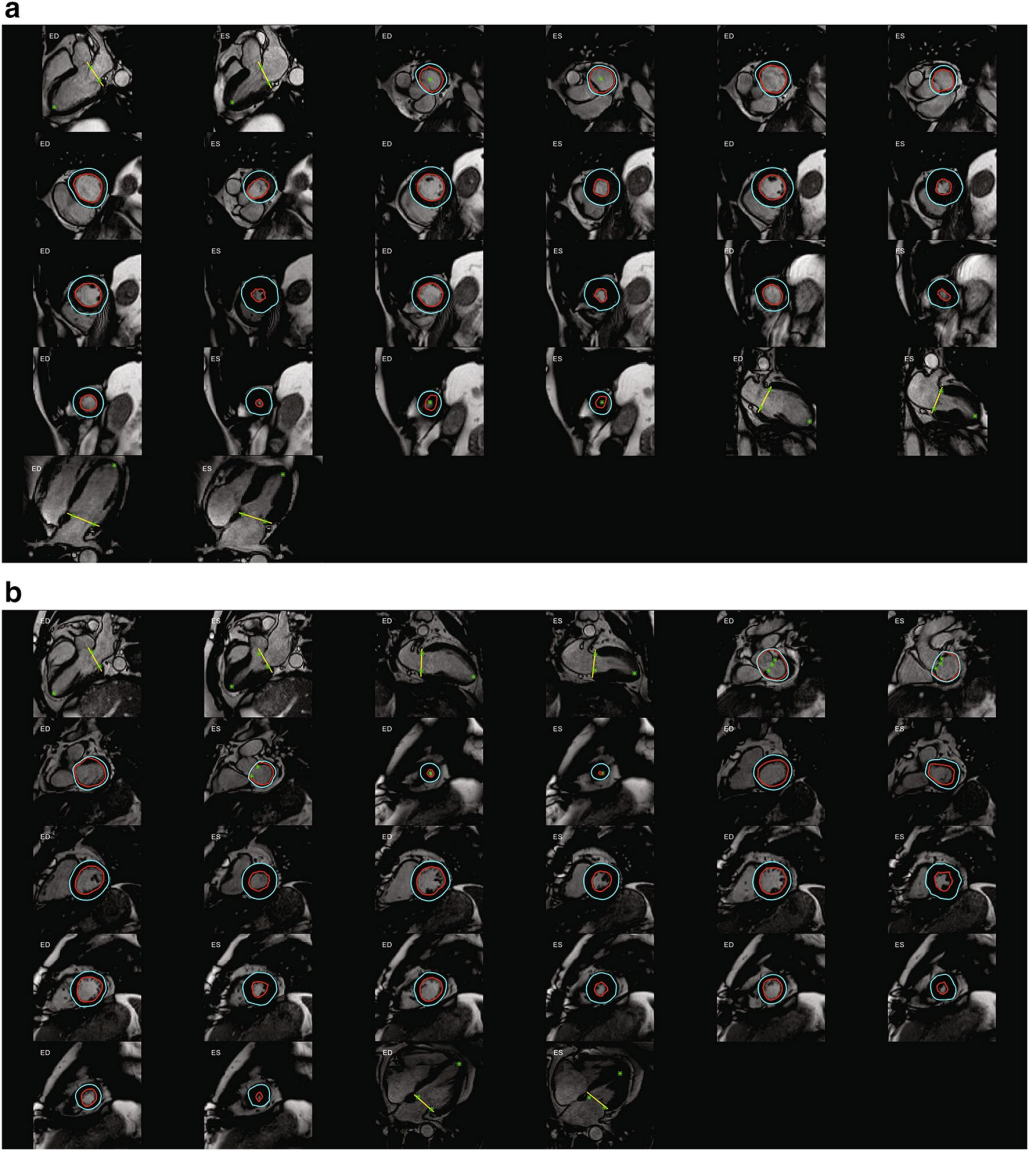
**a** InlineVF results for a typical case with good agreement for volume (<5 ml for EDV and ESV) but overestimation of mass (61 g) compared with manual analysis. **b** InlineVF results for a case with relatively large discrepancy between manual and inlineVF results (30 ml in EDV). Contours show errors at the* base slice*

Recently, fully-automatic analyses of ventricular function are becoming available, with immediate application to large cohort studies [10–13]. In the UK Biobank CMR imaging examination, the Siemens *syngo* InlineVF (Siemens Healthcare, Erlangen, Germany) fully automated analysis of left ventricular (LV) volume was performed during acquisition. This software automatically identifies LV landmarks at the LV base (mitral valve) and apex in long-axis cine acquisitions, locates endocardial and epicardial contours at ED and ES in each short-axis cine slice, and performs volume calculations to determine ventricular function parameters (Fig. 1). However, the software was designed for supervised analysis with visual assessment for quality control in a clinical setting. Since these results are already available to researchers as part of the initial UK Biobank CMR image dataset, their application to large cohort studies such as UK Biobank requires investigation. Although the D13A version was used in the initial automated analysis, a subsequent E11C version will also be made available. This paper compares the performance of these versions in the first 5,000 UK Biobank cases.

We sought firstly to evaluate the performance of automated ventricular function analysis against a standard manual analysis, in a substantial sub-cohort of UK Biobank. The second objective was to correct for bias between the automated and manual analyses to enable automated results to be used in future UK Biobank case-control studies.

## Methods

### Subjects

CMR examinations from the first 5065 UK Biobank imaging extension participants were assessed. All participants gave written informed consent and the appropriate institutional review boards approved the study protocol (National Research Ethics Service North West 11/NW/0382).

### Imaging protocol

The full CMR protocol and rationale have been described in detail previously [3]. Briefly, all imaging was conducted on a 1.5 T scanner (MAGNETOM Aera, *syngo* MR D13A, Siemens Healthcare GmbH, Erlangen, Germany) using a phased-array cardiac coil. Ventricular function scans consisted of retrospectively gated cine balanced steady-state free precession breath-hold acquisitions performed in horizontal long axis, vertical long axis, left ventricular outflow tract orientations, as well as a complete short axis stack covering the left and right ventricles. Typical parameters were: TR/TE = 2.6/1.1 ms, flip angle 80°, GRAPPA factor 2, voxel size 1.8 × 1.8 × 8 mm^3^ (6 mm for long axis). The actual temporal resolution of 32 ms was interpolated to 50 phases per cardiac cycle (~20 ms).

### Manual analysis

Manual analysis of LV volumes and mass were performed in accordance with the Society of Cardiovascular Magnetic Resonance recommendations [4]. Eight readers in two core laboratories were trained according to standard operating procedures prior to study commencement, to ensure minimal inter-observer bias. CMR examinations were analysed using cvi^42^ post-processing software (Version 5.1.1, Circle Cardiovascular Imaging Inc., Calgary, Canada). The ED frame was selected as the first frame of the series and the ES frame was selected as the smallest LV blood pool area in the mid-ventricular slice. At both ED and ES, the most basal slice included had at least 50% of the LV blood pool surrounded by myocardium. Papillary muscles were included in the blood pool. Inter-observer errors were quantified in 50 randomly selected cases. The software provided ED and ES volume (EDV, ESV respectively), ejection fraction (EF), stroke volume (SV) and mass (LVM). LVM was calculated assuming a myocardial density of 1.05 g/ml.

### InlineVF

The D13A version of the InlineVF analysis algorithm was performed as part of the image acquisition and results stored as a separate DICOM image series as part of the image data for each case. The InlineVF algorithm has been described previously [10, 14, 15]. Briefly, shortest path algorithms were used to determine epicardial and endocardial contours that were propagated to other frames and used in other slices as a geometric prior. All frames were segmented in each slice using an inverse consistent deformable registration to register all frames to the first frame. The segmentation was propagated to other frames through the forward and backward deformation fields. The long axis slices were used to detect basal and apical landmarks using machine learning methods [15]. These landmarks were used to define a base plane approximation at the level of the mitral valve, which was used to cut contours to avoid inclusion of atrial volume in the ventricle. Papillary muscles were included in the blood pool. LV volumes and mass were calculated by slice summation, with a correction for the location of the base plane. LVM was calculated by assuming a myocardial density of 1.05 g/ml. In this paper we used the LVM calculated at ED for both InlineVF and manual estimates.

A subsequent release of the software, version E11C, was applied retrospectively in a batch-processing mode prototype. The E11C version provided an estimate of LVM calculated from the epicardial contours, whereas the D13A version did not. Other changes incorporated in E11C included a refined detection of the LV blood pool: In addition to the detection of the heart based on a Fourier transform over time to detect moving objects, RV insert points were also detected to derive a blood pool feature point. Thresholded connected components were then clustered across slices to recover the blood pool, using information about the location of the connected component with respect to the blood pool point as an additional feature in the clustering algorithm. The batch-processing prototype was implemented on a Windows 7 workstation using Python and Windows Batch scripts. The input was the directory containing DICOM images for an entire study and the output was the LV mass and volume.

Outliers with EDV or ESV > 500 ml in the automated results were rejected as unphysiological. For the E11C results, a rapid visual assessment of resulting contours was also performed. Algorithm failures were identified if the InlineVF contours were grossly erroneous (e.g. contouring of organs other than the LV) or identification of the landmarks or LV base plane was grossly incorrect or absent.

### Statistics

Agreement was assessed by Bland–Altman analysis of bias (mean difference) and precision (standard deviations of the differences), 95% limits of agreement, and two-way random single measures intra-class correlation coefficient (ICC) for agreement (i.e. including systematic differences) [16]. The Levene test was used to test for differences in precision. Significant differences were defined at p < 0.05. Linear regression was performed to determine a correction between manual and InlineVF parameters. The correction parameters were assessed using Monte Carlo cross-validation [17]. The dataset was randomly divided into 90% training and 10% test cases, and prediction errors calculated in the test cases using the linear correction derived from the training cases. The resulting prediction errors were averaged for 500 trials. Statistical analysis was performed using R (version 3.3.2) statistical software [18].

## Results

A total of 5065 consecutive UK Biobank CMR examinations were evaluated. Of these, 191 cases had either CMR data of insufficient quality for manual LV analysis or the CMR identifier could not be matched with the UK Biobank identifier. Manual LV analysis was performed in the remaining 4874 cases. Table 1 shows participant demographics. Typical inter-observer errors quantified in 50 cases were −2.2 ± 4.7 ml for EDV, −2.4 ± 4.7 ml for ESV, 0.53 ± 5.8 ml for SV, 2.7 ± 6.6% for EF, 1.9 ± 6.5 g for LVM.

**Table 1:** Participant characteristics

n	4874
Age (years)	62 ± 8
Male	2313 (48%)
Caucasian ethnicity	4728 (97%)
Weight (kg)	76 ± 15
Height (cm)	170 ± 9
Body mass index (kg/m^2^)	26 ± 4
Body surface area (m^2^)	1.86 ± 0.21
Systolic blood pressure (mmHg)	137 ± 18
Diastolic blood pressure (mmHg)	79 ± 10
Heart Rate (bpm)	70 ± 12
Use of anti-hypertensive, lipid-lowering medications or insulin	1593 (33%)
Diabetes	256 (5%)
Cardiovascular diseases^a^	401 (8%)
Respiratory diseases^b^	805 (17%)
Renal diseases^c^	12 (0.2%)

All continuous values are reported in mean ± standard deviation (SD), while categories are reported in number (percentage)

^a^Angina, myocardial infarction, heart failure/pulmonary oedema, arrhythmia, cardiomyopathy, atrial fibrillation, stroke, ischaemic stroke, transient ischaemic attack, peripheral vascular disease

^b^Asthma, chronic obstructive airways disease, emphysema/chronic bronchitis, bronchiectasis, interstitial lung disease, asbestosis, pulmonary fibrosis, fibrosing alveolitis/unspecified alveolitis, sleep apnoea, respiratory failure;

^c^Renal failure, renal failure requiring dialysis, diabetic nephropathy

InlineVF D13A results were obtained in 4423 cases (9% failure rate). However, several cases returned erroneous volumes due to gross failures of the algorithm to detect LV features. Some of these cases could be readily identified as unphysiological EDV or ESV. However, many cases could not be automatically identified as failures from the volumes alone. Excluding the 10 cases with EDV or ESV > 500 ml as implausible, and so outliers for this cohort, comparisons between manual and automated results in the remaining 4413 cases are shown in Table 2. Bias (mean differences) were small but standard deviations of the differences were relatively large, leading to wide limits of agreement.

**Table 2:** Comparison of manual and inlineVF D13A results, n = 4413

	EDV (ml)	ESV (ml)	SV (ml)	EF (%)
Manual	144.0 ± 34.3	59.2 ± 20.3	84.9 ± 19.2	59.4 ± 6.4
InlineVF D13A	139.9 ± 37.5	62.3 ± 25.8	77.6 ± 18.2	56.1 ± 6.5
Differences	−4.2 ± 21.6	3.1 ± 19.0	−7.3 ± 10.9	−3.3 ± 6.0
Limits of agreement	(−46.6, 38.2)	(−34.1, 40.4)	(−28.7, 14.0)	(−15.0, 8.5)
R^2^	0.676	0.466	0.692	0.318
ICC	0.813	0.658	0.772	0.499

Values are mean ± SD. Unphysiological cases were removed

InlineVF E11C results were obtained in 4775 cases (2% failure rate). Excluding the 101 cases with EDV or ESV > 500 ml, comparisons between manual and automated results in the remaining 4674 cases are shown in Table 3. Biases were again small and precision in EDV and ESV was somewhat improved (p < 0.05) over the D13A version. LVM showed consistent overestimation relative to manual results.

**Table 3:** Comparison of manual and inlineVF E11C results, n = 4674

	EDV (ml)	ESV (ml)	SV (ml)	EF (%)	LVM (g)
Manual	144.3 ± 34.3	59.4 ± 20.4	85.0 ± 19.3	59.3 ± 6.4	89.7 ± 24.8
InlineVF E11C	141.8 ± 35.0	53.7 ± 22.8	88.1 ± 19.9	62.7 ± 7.3	119.2 ± 32.5
Differences	−2.5 ± 17.4	−5.6 ± 16.0	3.1 ± 11.0	3.4 ± 6.2	29.5 ± 17.8
Limits of agreement	(−36.6, 31.5)	(−37.0, 25.7)	(−18.4, 24.7)	(−8.8, 15.6)	(−5.4, 64.4)
R^2^	0.765	0.535	0.771	0.348	0.705
ICC	0.872	0.703	0.832	0.521	0.533

Values are mean ± SD. Unphysiological cases were removed

Rapid visual assessment of the 4775 InlineVF E11C contours and landmarks found 178 cases with gross errors in automated contour or landmark placement (36 in contours only, 46 in the landmarks only, and 96 in both contours and landmarks). Results for the remaining 4597 cases (6% total failure rate) are shown in Table 4. The precision in the EDV, ESV, SV and EF estimates were considerably improved (all p < 0.05), whereas LVM precision (p = 0.12) was unaffected, compared with Table 3.

**Table 4:** Comparison of manual and inlineVF E11C Results, n = 4597

	EDV (ml)	ESV (ml)	SV (ml)	EF (%)	LVM (g)
Manual	144.5 ± 34.3	59.4 ± 20.5	85.1 ± 19.2	59.3 ± 6.4	89.8 ± 24.8
InlineVF E11C	141.5 ± 33.1	53.1 ± 19.3	88.4 ± 19.1	63.0 ± 6.4	119.7 ± 32.0
Differences	−3.0 ± 11.6	−6.4 ± 9.0	3.4 ± 9.8	3.5 ± 5.1	29.9 ± 17.0
Limits of agreement	(−25.7, 19.7)	(−24.1, 11.3)	(−15.9, 22.7)	(−6.3, 13.7)	(−3.4, 63.2)
R^2^	0.887	0.807	0.754	0.466	0.725
ICC	0.937	0.853	0.855	0.586	0.534

Values are mean ± SD. Visually assessed failures were removed

**Fig. 2:**
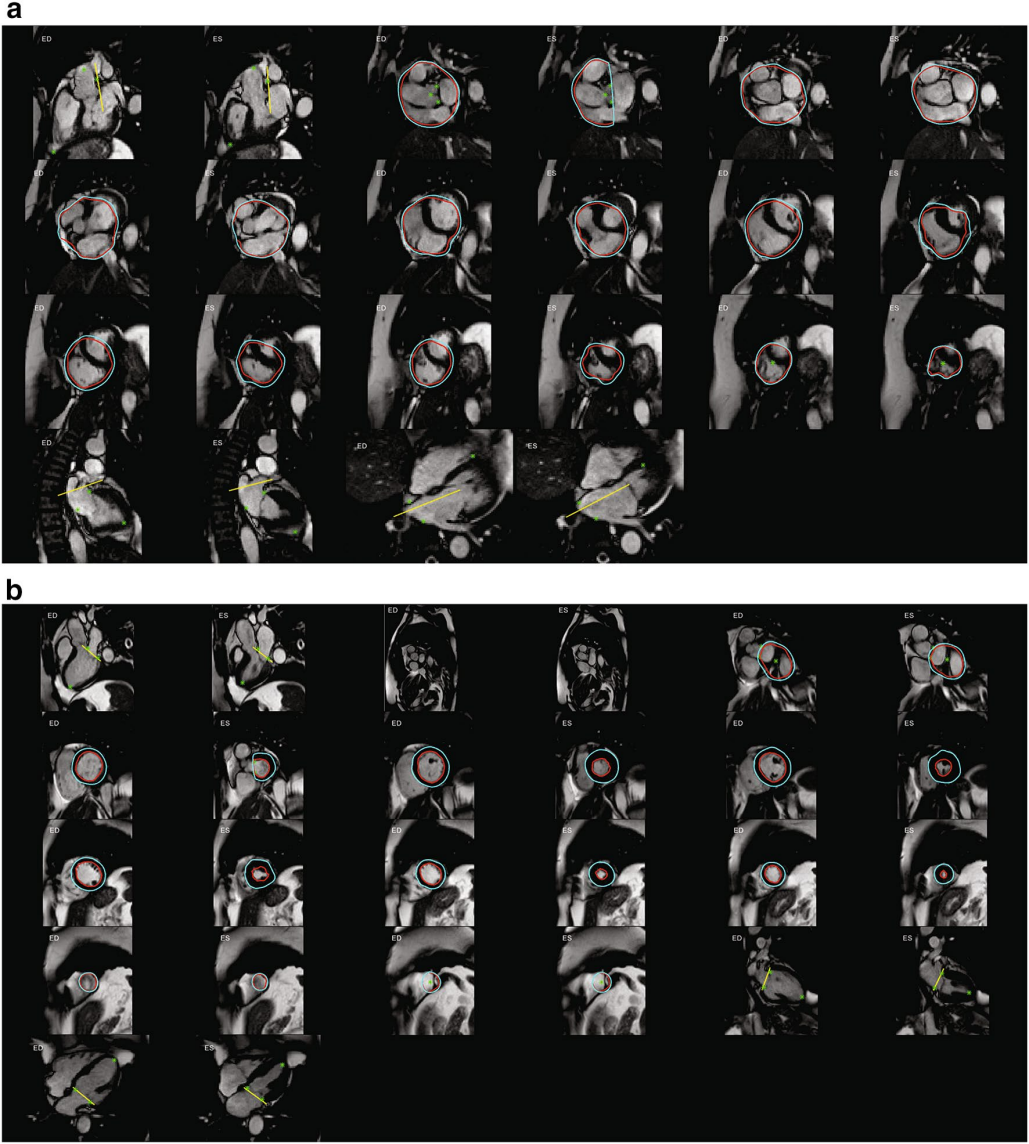
InlineVF results for two cases classified as failure by visual inspection. **a** Base landmarks incorrect and LV contours cover both ventricles. **b** LV contours show gross errors at the* base and apex*

Figure 1a shows an example of InlineVF E11C results for a typical case with good agreement for volume (<5 ml for EDV and ESV) but overestimation of mass (61 g) compared with manual analysis. This shows some errors in contour placement for the basal slice, but the difference in LVM was mainly due to consistently larger epicardial contours for all slices. Figure 1b shows a case with a relatively large discrepancy between manual and InlineVF results (30 ml in EDV). This case illustrates good contours for most slices, except for the basal slice. Figure 2a shows a case that was classified as a failure by visual inspection. The algorithm in this case has detected both ventricles as the LV. Figure 2b shows another case classified as a failure, but with errors at the basal and apical slices only.

**Fig. 3:**
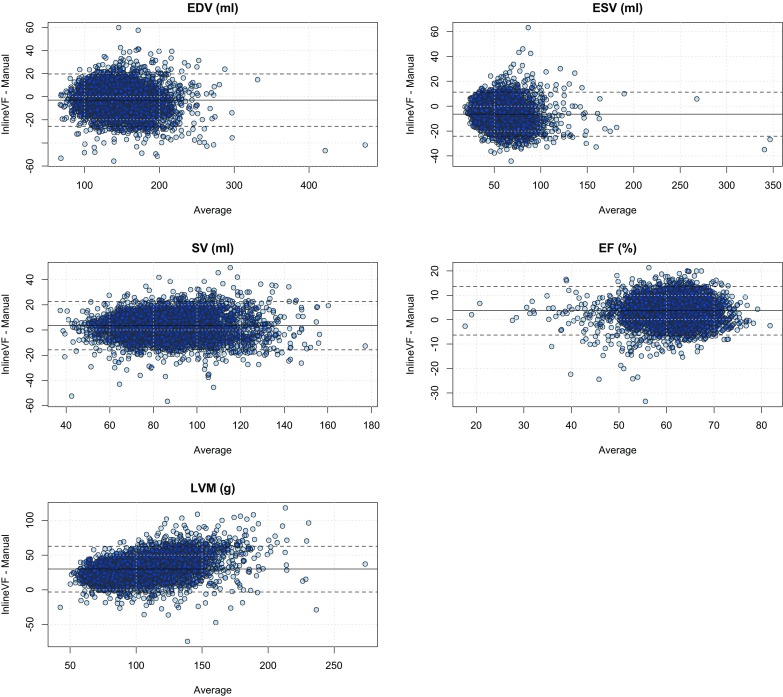
Bland–Atman plots for InlineVF E11C results with visual failures removed (n = 4597). *Dotted lines* are mean difference ± 1.96 SD

**Fig. 4:**
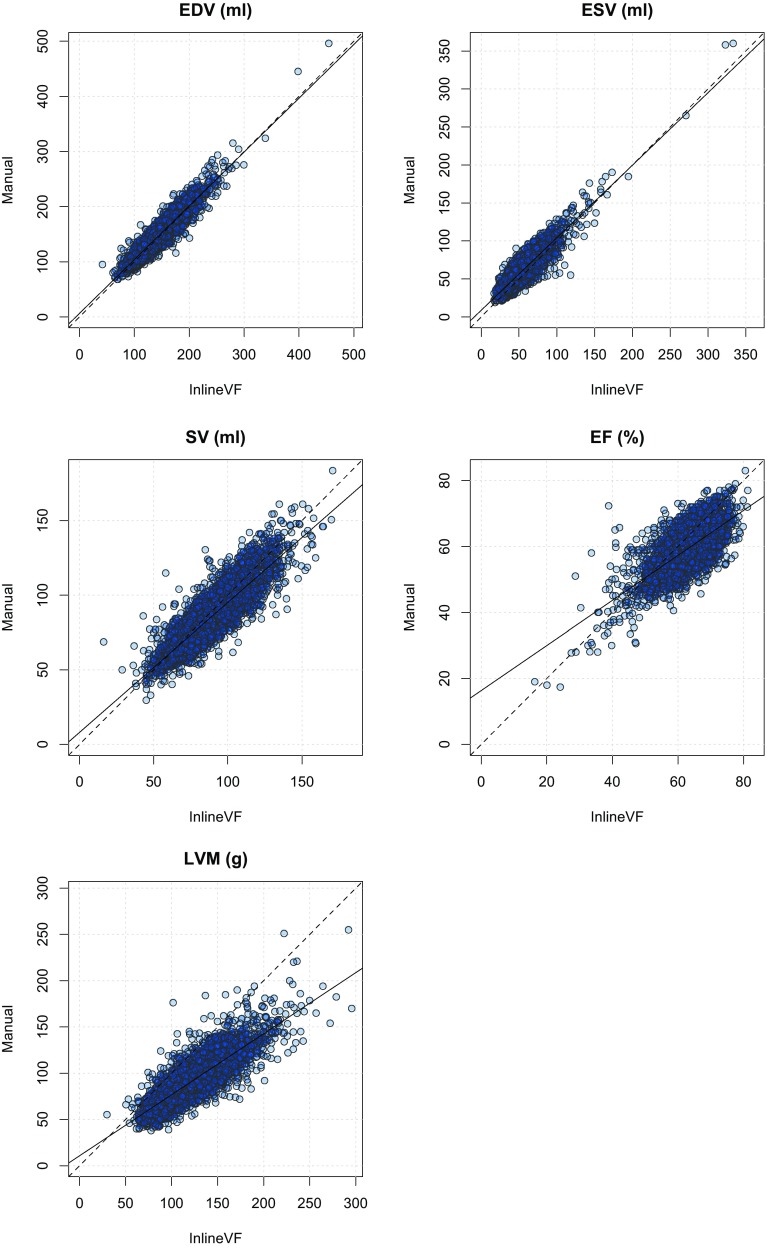
Linear regression plots the InlineVF E11C results with visual failures removed (n = 4597). *Solid line* is the linear regression; *dotted line* is the line of identity

Figure 3 shows Bland–Altman plots for ventricular function parameters and LV mass for the InlineVF E11C results with visual failures removed (n = 4597). LVM showed a consistent overestimation with the InlineVF results, increasing with increasing mass. This is verified in Fig. 4 (linear regression plots), in which LV EDV and ESV showed regression lines near the line of identity, and LVM had the largest deviation from identity, with a consistent overestimation that was well characterized by a linear regression.

A tenfold cross-validation was performed to determine the robustness of the linear regression parameters. The resulting regression parameters are shown in Table 5. Slopes were close to identity for EDV and ESV, but lower slopes and higher intercepts were found for SV, EF and LVM. Table 5 also shows the errors of prediction if automated results were used in place of manual results (after linear correction using the mean slope and intercept found by cross-validation). Bias has been removed, as expected, but precision is also improved for the LVM estimate. The inter-class correlation coefficients between the corrected automated results and the manual results also show improvement for all parameters.

**Table 5:** Linear regression results (Monte Carlo cross-validation)

	Slope	Intercept	Corrected errors	Corrected ICC
EDV	0.975 ± 0.003	6.53 ± 0.36 ml	0.0 ± 11.54 ml	0.940
ESV	0.954 ± 0.004	8.79 ± 0.22 ml	0.0 ± 8.99 ml	0.893
SV	0.870 ± 0.003	8.13 ± 0.26 ml	0.0 ± 9.52 ml	0.859
EF	0.682 ± 0.005	16.34 ± 0.30%	0.0 ± 4.68%	0.636
LVM	0.660 ± 0.003	10.84 ± 0.29 g	0.0 ± 13.01 g	0.841

Corrected errors are mean differences and standard deviation of differences between manual and corrected InlineVF values (using the average linear regression model from cross-validation). Similarly corrected ICC uses the corrected InlineVF values

In order to estimate the number of cases required for case control studies using the InlineVF estimates in UK Biobank, a number of assumptions are required. The error of the measurement can be estimated from the precision values shown in Table 5. Table 6 shows indicative power calculations illustrating the number of subjects required to detect a difference in CMR variables, assuming a type I error rate of 5%, and standardized effect size (mean effect divided by standard deviation) of 30–100%. For example, a study designed to detect a 30% standardized effect size for LV mass, assuming a standard deviation of 13 g (Table 5), would require 234 patients in each group to detect of a mean change of 4 g (30% of 13 g) with 90% power. However, additional variation is likely due to variability in the manual results and intrinsic biological variability.

**Table 6:** Example power calculations showing number of cases (in each group) required to detect a difference in CMR variables between two groups of equal sizes

Power	80%			90%		
Effect size	30%	60%	100%	30%	60%	100%
n	175	45	17	234	59	22

Significance level is 0.05, and a two-sided *t* test is assumed. Standardized effect sizes of 30, 60 and 100% are shown

## Discussion

Fully automated image analysis methods are desirable for large cohort studies such as UK Biobank, due to the complex nature of image analysis and the requirement for large numbers of cases. Automated analysis tools for LV function are now becoming more widely available, for a recent review see [11]; however, most studies have reported limited numbers of cases. An open benchmark challenge comparison of fully-automated and semi-automated methods in 95 cases showed that the fully-automated Siemens InlineVF algorithm performed as well as semi-automated methods [10]. More recently, automated methods have been reported in studies with over 1000 participants [13, 19].

The Siemens InlineVF analysis tool was one of the first fully automated LV analysis methods commercially available on standard scanners [20, 21], and the D13 version was enabled for the initial UK Biobank imaging acquisitions and these results are available to researchers as part of the initial image dataset. However, this tool was designed for clinical review in association with visual inspection of results, as required by regulatory and certification bodies. Application to epidemiological research studies such as UK Biobank is therefore unclear. In this study, we report the largest evaluation of a fully-automated LV analysis algorithm performed to date, to our knowledge. Improvements with the E11C version of InlineVF as well as LVM quantification, using visual inspection and linear bias correction, are demonstrated.

Although the detection failure rate was considerably improved in the E11C version, a review of remaining failures highlighted some conditions where misdetection was more likely. Firstly, the aorta can appear very bright and pulsate strongly in some cases, leading to mis-detection of the left ventricular blood pool. Secondly, the whole heart (left and right ventricles) may be detected as the left ventricle if the contrast between blood and myocardium is weak, or if there is some blurring due to irregular heart rate or breathing. Thirdly, the algorithm can fail if the gray level distributions of the different regions (blood, myocardium, lungs, partial voluming) cannot be modeled correctly due to unexpected intensities and contrast in the images. To some extent, such failures could be mitigated by re-acquisition with better breath-holds, adjusted slice positioning, or arrhythmia rejection. However, in the context of large cohort studies such as UK Biobank, it is not desirable to expend a large effort to achieve a 100% success rate, since a small number of drop-outs can be accommodated.

The best precision (standard deviation of the differences) obtainable was about twice that of the manual inter-observer precision for EDV and ESV, and over three times for LVM. However, the technology of automated image analysis is currently advancing at a rapid pace, with new developments in machine learning (e.g. deep convolutional neural networks) showing considerable promise [22]. Therefore, we expect that improvements in algorithms will lead to improved precision, leading to a reduction in the number of cases required for case-control studies.

Limitations of the study include the visual assessment required to detect algorithm failures. Although this is fast on a case-by-case basis, review of many thousands of cases is time-consuming. In the future it would be useful to automatically assess the quality of the analysis, for example to automatically flag failures, or give an uncertainty in the estimate. Some failures could be detected simply by implausibly large or small LV volumes (as in Fig. 2a). However this is not possible for the case in Fig. 2b, a more complex method is required. It may be possible to detect such failures using machine learning methods, which would in turn lead to better performance of the original detection. This is an active area of further study. Another area of future research is the correction of breath-hold misregistration. The long axis slices were used to determine a basal cut-off plane below which volume was included in the ventricle. Inconsistent breath-holding can influence the position of this plane. Although the base plane is an average of all the long axis slices, and is therefore robust to moderate breath-hold misregistation, future methods will enable better registration of the short and long axis slices.

Another potential limitation was that the manual results were treated as correct for all calculations. It is known that the manual contouring can show bias between centers due to differences in training [5]. Suinesiaputra et al. [5] provided a consensus dataset of 15 cases derived from analyses from seven independent centers for benchmarking purposes. Readers from the current study also analyzed these cases, resulting in typical consensus errors of EDV = −6.72 ± 12.03 ml; ESV = −3.58 ± 12.75 ml; EF = −0.72 ± 3.51%; LVM = −1.28 ± 11.96 g. Thus, the manual results of the current study are in good agreement with previous studies and other centers. Another source of potential error is the choice of ED and ES frames, since this was assessed manually by visual inspection of the mid-ventricular slice. The automatic algorithm, in contrast, computed volume for all frames and reported the maximum and minimum volumes. Future studies should investigate the performance of different vendor’s software and quantify differences between methods.

## Conclusions

Automated InlineVF results provided in UK Biobank can be used for case-control studies, provided visual assessment for quality control and linear adjustment of bias are performed. Further improvements in performance are expected in the near future with rapid advances in automated analysis technologies.

## Abbreviations

CMR: Cardiovascular magnetic resonance imaging
EDV: End-diastolic volume
ESV: End-systolic volume
SV: Stroke volume
EF: Ejection fraction
LV: Left ventricular
LVM: Left ventricular mass
SSFP: Steady state free precession
